# Association between estimated fluoride intake and dental caries prevalence among 5-year-old children in Korea

**DOI:** 10.1186/s12903-015-0153-0

**Published:** 2015-12-30

**Authors:** Min-Ji Kim, Han-Na Kim, Eun-Joo Jun, Jung-Eun Ha, Dong-Hun Han, Jin-Bom Kim

**Affiliations:** Department of Dental Hygiene, Division of Health Sciences, Dongseo University, Busan, South Korea; Department of Dental Hygiene, College of Health Sciences, Cheongju University, Cheongju, South Korea; Department of Preventive and Community Dentistry, School of Dentistry, Pusan National University, 49 Busandaehak-ro, Mulgeum-eup Yangsan-si, Gyeongsangnam-do 626-870 South Korea; Department of Dental Hygiene, Baekseok University, Cheonan, Chungnam South Korea; Department of Preventive and Social Dentistry, School of Dentistry, Seoul National University, Seoul, South Korea

## Abstract

**Background:**

The purposes of this study were to estimate the fluoride intake from food and drink in 5-year-old Korean children, and to measure the association between estimated fluoride intake and dental caries prevalence.

**Methods:**

The study involved a secondary analysis of raw data from the 4^th^ Korea National Health and Nutrition Examination Survey (KNHANES; 2007–2009). The study subjects were 167 boys and 147 girls aged 5 years who had undergone both physical and nutritional examination as part of the survey. The KNHANES comprised a health questionnaire, a physical examination, and a nutritional examination. The nutritional examination of KNHANES consisted of 3 parts: a dietary life survey, a food-frequency questionnaire, and a food intake investigation. The food intake investigation used the 24-h recall method, with information being provided by the children’s parents. On the basis of this information, we evaluated the fluoride content in a total of 310 food items using the hexamethyldisiloxane (HMDS)-facilitated diffusion method, modified using Taves’ microdiffusion method. As part of the KNHANES survey, oral examinations were conducted at a mobile examination centre by trained dentists using dental mirrors under a fluorescent light. These examinations were performed using methods proposed by the World Health Organization.

**Results:**

The dietary fluoride intake of 5-year-old Korean children was estimated to be 0.35 mg/day, or 0.016 mg/kg/day. The “decayed or filled surfaces” (dfs) indices of primary teeth were higher in children who had a lower dietary intake of fluoride. There was a significant inverse association between dietary fluoride intake and the prevalence of dental caries.

**Conclusion:**

The inverse association between dietary fluoride intake levels and prevalence of dental caries implies that the introduction of community caries prevention programmes may be beneficial. Such programmes would include water fluoridation and a fluoride supplementation programme.

## Background

Fluoride has been one of the most effective and widespread agents used to prevent dental caries [1]. It is currently considered a caries-preventive material decreasing the rate of enamel demineralisation and enhancing the remineralisation of early carious lesions [2–4].

Children are more vulnerable to dental caries than adults. Fluoride has been used to prevent dental caries [1]. When it comes to fluoride effects on dental caries, fluoride agents such as varnishes, gels and water fluoridation programme were covered as a main subject. The amount of fluoride intake hasn’t attracted attention. In children, the two most important sources of fluoride intake are the diet and dentifrice [5–9]. Fluoride may also be inhaled (cigarette smoking, industrial emissions), absorbed dermally (chemicals or pharmaceuticals), or ingested in the form of fluoride-containing drugs or soil [1]. Cardoso et al. [10] reported that fluoride intake via dentifrices was higher than that via diet. Miziara et al. [11] reported that fluoride intake via food was 11.8 %; via water, 17.2 %; via other beverages, 14.7 %; via dentifrices, 56.3 %. Their study was performed in an area where the water is fluoridated.

Most studies have measured dietary fluoride intake [8, 10–16], while several have reported intake via fluoride-containing dentifrices [17] or total fluoride intake [6].

There are two ways of estimating total dietary fluoride intake: the duplicate plate method, and dietary intake record [5]. The duplicate plate method requires the participant to prepare an exact duplicate of all foods and drinks (including water) ingested for one or more days [6]. Several studies have used this method to report the dietary fluoride intake of children [14, 18], and it is thought to be an accurate means of assessing fluoride and nutrient intake [18]. However, the duplicate plate method is difficult to conduct in children because it requires the compliance of parents. Furthermore, foods and drinks must be collected for analysis. This can raise ethical dilemmas, especially in socioeconomically disadvantaged countries. The duplicate plate method is therefore not suitable for broad epidemiological surveys [18]. As an alternative then, fluoride intake can be estimated on the basis of recorded diets. Most dietary surveys use this approach, which involves itemising all foods and drinks which were ingested during one or more days, as well as the quantities thereof [5]. The records are usually written, although an additional interview should always be conducted [5].

The percentage of Korean population covered by water fluoridation programme that started in 1981 decreased from 12.7 to 6.1 % between 2000 and 2012. Jin BH et al. [19] reported early children caries (ECC) of Korean children in 2003 that the mean dmft index of 48–59 month-old children was 6.92 and the percentage of children with ECC was 90.8 %. The national mean DMFT score in 6-year-old was 0.26 in 2000 and 0.22 in 2006 [20]. Lee HJ et al. [21] analysed risk factors of dental caries in childhood with a five-year survival analysis and reported that dental caries at an individual level can be associated with the experience of dental caries in primary teeth.

The Korea National Health and Nutrition Examination Survey (KNHANES) is a study which assesses the health and nutritional status of adults and children in Korea [22]. The survey combines interviews and physical examinations. The intake of nutrients other than fluoride has been reported using daily food intake information from the KNHANES [22]. However, no reports have yet used the KNHANES to investigate the fluoride content of the foods consumed in Korea. It is therefore needed to provide sound advice regarding an adequate fluoride intake level in children, and to accurately estimate the total current dietary fluoride intake. The latter objective can be achieved by conducting national investigations into the fluoride content of foods.

The aims of the study were to 1) estimate the fluoride intake from foods and drinks in 5-year-old Korean children, 2) to measure the association between the estimated fluoride intake and dental caries prevalence on primary dentition and to recommend adequate fluoride intake by water fluoridation programme and fluoride supplements.

## Methods

### Data source and study population

This study involved a secondary analysis of raw data from the 4^th^ KNHANES (2007–2009). The Korea Centres for Disease Control and Prevention at the Ministry of Health and Welfare approved the research plan and the use of the data. Ethical clearance of the 4^th^ KNHANES was approved by the Institutional Review Board of Korea Centres for Disease Control and Prevention (approval number: 2007-02CON-04-P, 2008-04EXP-01-C, 2009-01CON-03-2C). Written consents were obtained from parents/guardians.

**Fig. 1:**
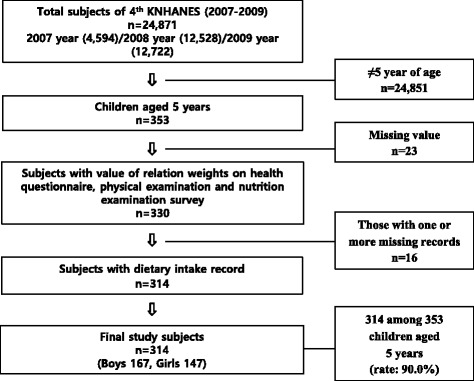
Flow diagram of subject selection process

The KNHANES, a nationwide survey which obtains national-level health statistics, ensured that the samples were representatives of the population by selecting subjects using a probability sampling method [22]. Briefly, based on the census of 2005, survey areas (neighbourhoods/townships/towns) were sampled using proportional allocation. From the selected areas, sampling units were extracted: 100 units in 2007, 200 units in 2008 and 200 units in 2009 survey. The 4^th^ KNHANES began in July 2007 and ended in December 2009. The dataset comprised 3 parts: a health questionnaire, a physical examination, and a nutritional examination [22]. The survey included 24,871 people over 1 year of age from 13,800 households (response rate: 78.4 %). Of these participants, 23,632 participated in both the health questionnaire and the physical examination (response rate: 74.5 %), and 22,137 participated in the nutritional examination (response rate: 81.8 %) [22]. All 5 year-old children were subjected. The subjects of our study were 167 boys and 147 girls aged 5 years who had participated in both the physical and nutritional examinations as part of the 4^th^ KNHANES. The subjects are shown in Fig. 1.

### The nutritional survey and oral examination

The nutritional survey of KNHANES was conducted via individual interviews which were carried out by professional researchers at home visits, and which took place a week after completion of the health questionnaire and physical examination. The nutritional survey was divided into three parts: a dietary life survey, a food-frequency questionnaire, and a food intake investigation [23]. The food intake investigation was conducted using the 24-h recall method, with information being provided by the children’s parents. The 24-h recall may be useful for validating other dietary assessment methods among populations with limited motivation or literacy [24]. Oral examinations were conducted at a mobile examination centre by four trained dentists using dental mirrors under a fluorescent light. Before the oral examination, educations for calibration were carried out for 4 days with intra-oral pictures and the simulation patients. Concordance rate of interclass was 0.92. These examinations were performed using methods proposed by the World Health Organization [25].

### Analysis of the fluoride concentration of food

#### Preparation of food samples

We analysed a total of 310 food samples using data from all the children. The samples were classified into 18 groups (e.g., cereals, vegetables, meat etc.). The foods most often ingested were obtained commercially, and other samples were selected at our convenience. To avoid using locally-grown or produced foods as experiments sample, foods which could be bought easily in Korea were used in this study. We used standard fluoride solutions of 0.01, 0.1, and 10 ppm. With these, we used sample masses of 0.01, 0.05, and 0.1 g. We confirmed that an increase in sample size from 0.01 to 0.1 g corresponded to an increase in concentration from 0.01 ppm to 10 ppm. As such, we ensured the precision of our results. After this standardisation process, we analysed various foods known to be low in fluoride, such as pasta, celery, hardtack and apple-juice. We were unable to find any significant differences despite using different sample masses. However, in foods known to be high in fluoride, such as kelp and seaweed, an approximately 10-fold increase in fluoride concentration was found when the sample mass was increased from 0.01 to 0.1 g. Thus, 0.1 g was determined to be a suitable sample mass. This ensured concentrations high enough for detection by the fluoride ion electrode, while minimising the error due to evaporation. Additionally, there were no significant differences found between the fluoride concentration values of foods kept in 0.1 g slices and those kept whole. Because the fluoride concentration was measured in many samples, it was necessary to minimise diffusion time in order to increase the efficiency of the study. The fluoride ion recovery rate at 6, 24, and 48 h was 86–97 %. The recovery rate measured at 6 h was the lowest, and over 48 h, the solute had disappeared, and as the diffusion time increased, the recovery rate did not increase. Accordingly, the diffusion time was set at 24 h in the reference [26].

### Measurement of fluoride content and estimated fluoride intake

In order to measure fluoride content in the food samples, it was necessary to separate fluoride that was chemically bonded to other components. We therefore used the hexamethyldisiloxane (HMDS)-facilitated diffusion method [26], modified using Taves’ micro diffusion method [27], to assay foods. Food samples of exactly 0.1 g were prepared. Each sample was placed at the bottom of a petri dish along with 2 ml of distilled water. Fifty μl of 0.05 N NaOH were dropped onto the inside of the petri dish cover. Petroleum jelly (Vaseline®, Sungkwang pharm. Co. Ltd., Cheonan, Korea) was applied to the inside periphery of the cover before the cover was sealed to the plate. Two millilitres of 3 N H_2_SO_4_-HMDS solution were injected into the small hole of the petri dish cover, and the hole was sealed using Vaseline®. The samples were shaken to allow diffusion, and the diffusion reaction was allowed to proceed for 24 h at room temperature. The drops of 0.05 N NaOH were then collected from the petri dish cover, and the drops of 25 μl of 0.2 N acetic acid were added. The final volume was adjusted to 100 μl using distilled water. 10 μl of TISAB III solution (Orion 940911, Orion ionplus® Application Solution, Thermo Fisher Scientific Inc., Beverly, MA, USA) was added and the fluoride concentration was measured using the fluoride ion electrode (Orion 9609 BNWP, Thermo Fisher Scientific Inc., Beverly, MA, USA). The standard solutions of 0.01, 0.1, 1.0, and 10 ppm were prepared by diluting a fluoride ion standard solution (100 ppm) accordingly. First, 5 ml of TISAB III solution was put in each standard solution of 50 ml, then the fluoride ion electrode was placed in the solution, and calibration was conducted while stirring at a regular speed within the range wherein no bubbles were formed. The fluoride content of each sample was measured 3 times, and the mean value was considered the true value.

The daily fluoride intake was estimated based on the results of each sample by multiplying the fluoride content of each food by the daily intake. The following equations were used:

$$ \mathrm{Daily}\ \mathrm{fluoride}\ \mathrm{intake}\ \left(\upmu \mathrm{g}/\mathrm{day}\right)\ \mathrm{per}\ \mathrm{per}\mathrm{son} = \left(\mathrm{fluoride}\ \mathrm{content}\ \mathrm{of}\ \mathrm{food}\ \mathrm{sample}\ \left[\left(\upmu \mathrm{g}/\mathrm{g}\right)\right]\right) \times \left(\mathrm{daily}\ \mathrm{intake}\ \mathrm{of}\ \mathrm{sample}\mathrm{d}\ \mathrm{food}\ \left[\mathrm{g}\right]\right) $$

$$ \mathrm{Fluoride}\ \mathrm{intake}\ \mathrm{per}\ \mathrm{kilogram}\ \mathrm{per}\ \mathrm{day}\ \left(\upmu \mathrm{g}/\mathrm{kg}/\mathrm{day}\right) = \left(\mathrm{daily}\ \mathrm{fluoride}\ \mathrm{intake}\ \mathrm{per}\ \mathrm{per}\mathrm{son}\left[\upmu \mathrm{g}\right]\right)/\left(\mathrm{body}\ \mathrm{weight}\kern0.5em \left[\mathrm{B}\mathrm{W}\right]\kern0.5em \mathrm{of}\ \mathrm{per}\mathrm{son}\kern0.5em \left[\mathrm{kg}\right]\right) $$

The reliability of the results was confirmed by comparing the results from the samples with those obtained from the same samples by means of intra-class correlation coefficients. The inter-class correlation coefficient measured using 60 of the 310 food items was 0.997, which is a very high value. The degree of conformity is measured by comparing the measured fluoride concentrations with those of second samples of the same food item. We measured this value using 17 of the 310 food items, and it was found to be 0.961, which is also a very high value.

### Statistical analysis

Using data from the 4^th^ KNHANES, we found out all daily intake food items of 5-year-old Korean children who had participated in the nutrition survey. We then measured the fluoride ion concentration of those foods in order to calculate the daily dietary fluoride intake per person, and the fluoride intake per kg of BW. PASW Statistics 18.0 (SPSS Inc., Chicago, IL, USA) was used for statistical analysis of the data. A p-value < 0.05 was considered significant. All analyses took into account the complex sampling design. In order to check the inter-investigator reliability of the fluoride ion concentration measurement, the intra-class correlation coefficients were calculated, and a reliability test was then performed. Gender, caries experience (number of dt and dft), BW and fluoride ion concentration were used as variables. We compared between the genders daily dietary fluoride intake, fluoride intake per kg of BW, both “decayed primary teeth” (dt) and “decayed or filled primary teeth” (dft) index using the general linear model. We compared between the genders active “decayed” (d) and “decay or filling” (df) rates of primary teeth using the chi-square test. We used the general linear model to find associations between daily dietary fluoride intake and dental caries prevalence.

## Results

Of the food groups consumed by children aged 5 years, vegetables were the most common with 66 among 310 items, followed by cereals with 44 items, then fish and shellfish with 41 items. Seaweed had the highest median fluoride concentration with 1.00 ppm, followed by other vegetation and with 0.71 ppm, and nuts with 0.47 ppm. Fish and shellfish had the highest mean fluoride concentration with 8.84 ppm, followed by seaweed with 2.85 ppm (Table 1). Table 2 shows the mean estimated daily dietary fluoride intake from each of the various food groups. This was not significantly different between gender groups. The highest estimated daily fluoride intake came from fish and shellfish, followed by seaweed.

**Table 1:** Fluoride concentration in various food groups (ppm)

Characteristics	Food groups	Items	Fluoride concentration	
			Median	Mean ± SE
Vegetable foods^a^	Cereals	44	0.18	0.24 ± 0.04
	Potatoes	7	0.36	0.43 ± 0.13
	Saccharide	14	0.12	0.21 ± 0.07
	Pulses	12	0.20	0.22 ± 0.05
	Nuts	9	0.47	0.47 ± 0.18
	Vegetables	66	0.09	0.19 ± 0.04
	Mushrooms	6	0.04	0.09 ± 0.03
	Fruits	27	0.04	0.12 ± 0.03
	Seaweed	7	1.00	2.85 ± 1.85
	Beverage	12	0.12	0.18 ± 0.05
	Seasoning	29	0.11	0.52 ± 0.18
	Vegetable oil	11	0.04	0.07 ± 0.03
	Other vegetation	4	0.71	0.84 ± 0.40
Animal foods^b^	Meat	11	0.09	0.15 ± 0.04
	Eggs	2	0.11	0.11 ± 0.08
	Fish and shellfishes	41	0.41	8.84 ± 5.66
	Oil and fats	7	0.11	0.13 ± 0.04
	Animal fats and oil	1	0.06	0.06

^a^Total number of items: 248

^b^Total number of items: 62

**Table 2:** Mean estimated daily dietary fluoride intake from various food groups (μg/day)

Characteristics	Food groups	Total	Male	Female	p-value^*^
Vegetable foods	Cereals	41.04 ± 1.29	42.34 ± 1.78	39.50 ± 2.07	0.325
	Potatoes	15.06 ± 1.54	14.49 ± 2.29	15.68 ± 2.01	0.696
	Saccharide	0.82 ± 0.13	0.69 ± 0.11	0.98 ± 0.25	0.276
	Pulses	5.94 ± 0.69	6.81 ± 1.00	4.86 ± 0.96	0.166
	Nuts	0.68 ± 0.36	0.96 ± 0.64	0.32 ± 0.08	0.323
	Vegetables	15.39 ± 1.36	16.49 ± 2.04	14.12 ± 1.75	0.376
	Mushrooms	0.41 ± 0.06	0.46 ± 0.08	0.35 ± 0.07	0.302
	Fruits	17.03 ± 2.32	19.05 ± 3.67	14.67 ± 2.57	0.326
	Seaweed	46.13 ± 11.67	45.86 ± 13.72	46.45 ± 19.99	0.981
	Beverage	34.76 ± 7.02	39.97 ± 10.72	27.80 ± 8.28	0.383
	Seasoning	7.69 ± 0.61	8.32 ± 0.80	6.95 ± 0.63	0.100
	Vegetable oil	0.20 ± 0.04	0.24 ± 0.06	0.16 ± 0.03	0.256
	Other vegetation	0.38 ± 0.11	0.18 ± 0.05	0.49 ± 0.16	0.063
Animal foods	Meat	5.61 ± 0.53	5.95 ± 0.85	5.24 ± 0.64	0.517
	Eggs	1.10 ± 0.10	1.31 ± 0.15	0.86 ± 0.11	0.013
	Fish and shellfishes	227.40 ± 41.61	280.03 ± 69.03	166.82 ± 43.31	0.171
	Oil and fats	13.36 ± 0.90	13.11 ± 1.17	13.66 ± 1.28	0.743
	Animal fats and oil	0.17 ± 0.05	0.11 ± 0.03	0.24 ± 0.09	0.161
Total daily estimated fluoride intake	μg/day	346.12 ± 37.54	393.95 ± 60.53	289.40 ± 41.77	0.163
	μg/kg/day	16.65 ± 1.76	18.57 ± 2.73	14.36 ± 2.13	0.231

The values indicate Mean ± SE

^*^Using the complex samples general linear model

The total estimated daily fluoride intake of the children studied was 346.12 μg/day. The total daily intake per kg of BW was 16.65 μg/kg/day (Table 2). The total daily fluoride intake of the male group was higher than that of the female, but this did not constitute a significant difference.

There were no significant differences in BW, df rate, dfs index, dfs index of anterior or posterior primary teeth between the male and female groups (Table 3). The dfs indices of children with a higher dietary fluoride intake were lower than those of children with a lower dietary fluoride intake. This inverse association was found to be statistically significant using complex samples general linear model (Table 4).

**Table 3:** Body weight, df rate, dfs index, dfs index of anterior and posterior primary teeth among 5-year-old children from the 4^th^ KNHANES

Variables	Total	Male	Female	p-value
Body weight (kg)	20.68 ± 0.25	20.98 ± 0.34	20.31 ± 0.32	0.124 ^a^
df rate (%)^b^	55.7	53.7	58.1	0.494^c^
dfs index^d^	4.89 ± 0.49	4.22 ± 0.55	5.57 ± 0.84	0.195^a^
dfs index of anterior teeth^e^	1.24 ± 0.26	1.03 ± 0.21	1.44 ± 0.48	0.442^a^
dfs index of posterior teeth^f^	3.66 ± 0.34	3.19 ± 0.43	4.13 ± 0.55	0.195^a^

The values indicate Mean ± SE, with the exception of df rate

^a^Using the complex samples general linear model

^b^Percentage of population affected with dental caries on primary teeth

^c^Using the complex samples chi-square test

^d^Mean number of decayed or filled surfaces of primary teeth

^e^Mean number of decayed or filled surfaces of anterior primary teeth

^f^Mean number of decayed or filled surfaces of posterior primary teeth

**Table 4:** Associations between the estimated dietary fluoride intake and the prevalence of dental caries

Dependent variables	Independent variables	β	SE	p-value (95 % CI^*^)^a^
dfs index^b^	Daily fluoride intake	-0.001	<0.001	<0.001 (-0.002, 0.000)
	Daily fluoride intake per kg of body weight	-0.023	0.006	<0.001 (-0.035,-0.011)
dfs index of anterior teeth^c^	Daily fluoride intake	0.000	<0.001	0.002 (-0.001, 0.000)
	Daily fluoride intake per kg of body weight	-0.009	0.003	0.005 (-0.014,-0.003)
dfs index of posterior teeth^d^	Daily fluoride intake	-0.001	<0.001	0.007 (-0.001,0.000)
	Daily fluoride intake per kg of body weight	-0.014	0.005	0.002 (-0.024,-0.005)

^*^95 % Confidence Interval

^a^Using the complex samples general linear model

^b^Mean number of decayed or filled surfaces of primary teeth

^c^Mean number of decayed or filled surfaces of anterior primary teeth

^d^Mean number of decayed or filled surfaces of posterior primary teeth

## Discussion

The prevalence of dental caries in developed countries has declined over the past several decades, and this is considered to be the result of the widespread use of fluoride from various sources [28]. Thus, topical exposure to even low levels of fluoride contributes to caries prevention, and is beneficial to the population at all ages [2, 3]. That said, in cases of excessive fluoride ingestion during the period of enamel formation, enamel fluorosis has been known to occur [28, 29]. Despite this, the optimal fluoride intake is not known accurately [4].

In this study, we estimated the dietary fluoride intake of Korean children using research data from the 4^th^ KNHANES. The intake was estimated to be 0.35 mg/day, or 0.016 mg/kg/day. In a similar study using the “recording dietary intake” method, Ophaug et al. [12] reported that the fluoride intake of children between the ages of 6 months and 2 years in the United States was 0.21–0.62 mg/day. Also, Schamschula et al. [13] reported that the fluoride intake of Hungarian children aged 4 years was 0.22–0.72 mg/day. In a more recent study, Miziara et al. [11] reported that the fluoride intake of Brazilian children aged 2–6 years was 0.48 mg/day.

Using the “duplicate diet” method, Chowdhury et al. [30] reported that the fluoride intake of children in New Zealand aged 11–13 months was 0.08–0.26 mg/day. Zohouri and Rugg-Gunn [31] reported that 4-year-old Iranian children had a fluoride intake of 0.39 mg/day. Yang et al. [14] reported that the fluoride intake of Korean children aged 2–4 years was 0.65 mg/day. Murakami et al. [18] reported that Japanese children aged 3–5 years had a total fluoride intake of 0.28–0.30 mg/day. In more recent studies, Oganessian et al. [8] reported that the fluoride intake of Czech children with a mean age of 4.75 years was 0.38 mg/day. Burt and Eklund [32] suggested that the optimal daily fluoride intake is 0.05–0.07 mg/kg/day. The dietary fluoride intake of children in this study was lower than the recommended optimal amount. However, the contribution of other fluoride sources was not accounted, thus still now daily fluoride intake of children might not be measured exactly.

The study of Yang et al. [14] quantitatively and qualitatively duplicated all foods ingested during 24 h. The food samples were homogenized and ashed, and the fluoride therein was isolated using the HMDS-diffusion method. The fluoride ion concentration was measured using an ion-specific combination fluoride electrode. The fluoride concentrations found in foods by Yang et al. [14] appeared to differ a little from those found in our study. Furthermore, our results were generally similar to those of the Food Value study of 2009 [33]. However, certain food items showed large differences. For example, the fluoride concentration of bananas found in the Food Value study [33] was 2.20 μg/100 g, whereas a value of 2.92 μg/100 g was found in our study. The fluoride concentration of strawberries in the Food Values study [33] was 4.00 μg/100 g, while it was 43.23 μg/100 g in this study. It is important to note at this point that the Food Values study [33] did not measure the fluoride content using Korean foods, but rather the foods of other countries. Levy et al. [34] advanced the criticism that large differences are observed in the measured fluoride content, even among similar food items. This may be caused by differences in seeds, soil, feed, maturity, growth environment, and among individuals. Thus, if any final standard tables of the fluoride content of foods are to be created, the results of this study may be more relevant than those of the 2009 Food Values study [33].

The Korean Nutrition Society [35] has suggested an adequate fluoride intake of 0.8 mg/day, and a tolerable upper fluoride intake of 1.7 mg/day in children aged 3–5 years; as well as an adequate fluoride intake of 2.0 mg/day, and a tolerable upper fluoride intake of 10 mg/day in children aged 9–11 years. However, these recommended amounts are not based on sufficient evidence, as there have been an insufficient number of systematic studies regarding fluoride intake. The values depend on reports from other countries to suggest recommended dietary allowances [8, 11, 13, 14, 18, 30–32]. This study provides valuable data; necessary for informing the recommended dietary fluoride allowances based on fluoride intake measurements from Korean foods in children who are highly likely to develop dental caries. Although this study was performed on a limited pool of children – those aged 5 years – additional similar studies may make it possible to recommend evidence-based dietary fluoride allowances. Specifically, studies regarding the foods that Koreans consume and involving other age groups may be useful. It is also necessary to investigate fluoride use within the body.

The total amount of daily fluoride intake in the male group was higher than that in the female, but this did not constitute a significant difference. The dfs indices of children with lower intakes of dietary fluoride were higher than those in children with higher intakes of dietary fluoride. This inverse association was statistically significant. Warren et al. [36] reported that children with dental caries generally had slightly lower fluoride intake, whereas those without dental caries or with dental fluorosis generally had slightly higher intake.

It is difficult to measure or assume the total daily fluoride intake. Many sources of fluoride intake such as tap water, fluoride containing-toothpaste, foods and others can contribute to total daily fluoride intake for children. A previous study sought to compare total daily fluoride intake according to fluoride concentrations in tap water reported that Foods made the largest contribution (63.9 %) to total daily fluoride intake [37]. In this study, seaweed, fish and shellfishes had higher fluoride concentration compared to the other foods. Therefore, it is needed to be analysed dental caries experience of children by living regions such as seaside and mountainside. Kim JY et al. [38] reported that in 2003 Korea, almost 97 % people had used fluoride containing-toothpastes. Fluoride containing- toothpaste are commonly used in Korea. Even though fluoride containing- toothpastes are considered as major source of fluoride intake for children, it could be assumed that there might not be significant differences of amount of fluoride intake by tooth paste among Korean children. Water fluoridation programme is implemented only for 6.1 % Korean people in 2012. Thus, fluoride in tap water could not be a potential confounding variable in this study.

Even though this study has valuable merit as necessary data for informing the recommended dietary fluoride allowances, it had some limitations. Firstly, the study used representative food samples. The food samples used in this study will have been different from the foods consumed by the subject children. The second limitation is the method of analysing the food samples. In the case of trace minerals such as fluoride, small errors in the analysis method may cause large differences in the estimated total ingested amount. In order to overcome such limitations, the measurement of fluoride concentration in each food was repeated in this study, in an attempt to minimise the differences. To promote further study into the anti-cariogenic effect of fluoride, it would appear to be necessary to check the amount of fluoride in foods using food tables. The fluoride intake via other products (toothpaste, fluoride supplements) should be studied as well. Research into the rate at which fluoride is discharged from the body is also necessary to establish a monitoring system of fluoride intake and discharge. To give the information for dietary fluoride intake recommendation, a study which analyse the fluoride intake effects according to the caries risk levels is needed to be conducted. The last limitation is confounding factors such as socioeconomic and oral health behavioural factors were not considered. Although there were confounding factors which were not considered in this study, the subjects of present study were driven from national oral health data and analysed using complex sampling method. It could be assumed that potential effects of socioeconomic and oral health behavioural factors might be offset, however, further study adjusting for confounding factors were needed and long-term cohort study is needed to confirm the relationship between fluoride intake and experienced dental caries in Korean children.

The higher prevalence of dental caries among children of lower dietary fluoride intake implies that the introduction of community caries prevention programmes should be considered. Such programmes would include water fluoridation and fluoride supplementation.

## Conclusions

In the 5-year-old Korean children studied, the estimated dietary fluoride intake via food was lower than the widely accepted optimal intake of fluoride, which is between 0.05 and 0.07 mg/kg of BW. Even confounding factors was not considered, the inverse association found herein between dietary fluoride intake and prevalence of dental caries implies that the introduction of community caries prevention programmes may be beneficial. Such programmes would include water fluoridation and fluoride supplementation.

## Abbreviations

KNHANES: Korea National Health and Nutrition Examination Survey
HMDS: hexamethyldisiloxane
BW: body weight

## References

[1] Burt BA (1992). The changing patterns of systemic fluoride intake. J Dent Res..

[2] Arends J, Ten Cate JM (1981). Tooth enamel remineralization. J Crystal Growth..

[3] Featherstone JD, Glena R, Shariati M, Shields CP (1990). Dependence of in vitro demineralization of apatite and remineralization of dental enamel on fluoride concentrations. J Dent Res..

[4] Levy SM (1994). Review of fluoride exposures and ingestion. Community Dent Oral Epidemiol..

[5] Clarkson J, Watt RG, Rugg-Gunn AJ, Pitiphat W, Ettinger RL, Horowitz AM (2010). Proceedings: 9th World Congress on Preventive Dentistry: Community participation and global alliances for lifelong oral health for all, Phuket, Thailand, September 7–10, 2009. Adv Dent Res..

[6] Guha-Chowdhury N, Drummond BK, Smillie AC (1996). Total fluoride intake in children aged 3 to 4 years–a longitudinal study. J Dent Res..

[7] Cressey P, Gaw S, Love J (2010). Estimated dietary fluoride intake for New Zealanders. J Public Health Dent..

[8] Oganessian E, Ivancakova R, Lencova E, Broukal Z (2011). Alimentary fluoride intake in preschool children. BMC Public Health..

[9] Buzalaf MA, Rodrigues MH, Pessan JP, Leite AL, Arana A, Villena RS (2011). Biomarkers of fluoride in children exposed to different sources of systemic fluoride. J Dent Res..

[10] Ophaug R, Singer L, Harland B (1985). Dietary fluoride intake of 6-month and 2-year-old children in four dietary regions of the United States. Am J Clin Nur..

[11] Schamschula RG, Duppenthaler JL, Sugar E, Un PS, Toth K, Barmes DE (1988). Fluoride intake and utilization by Hungarian Children: associations and interrelationships. Acta Physiol Hung..

[12] Yang SJ, Moon HS, Paik DI, Kim JB (2002). A study on the fluoride intake from routine diets. J Korean Acad Oral Health..

[13] Han DH, Lee UJ, Kim DH, Kim MJ, Hwang SJ, Kim JB (2010). Evaluation of fluoride concentration in tea drink and estimation of daily fluoride intake by tea drink in Korea. J Korean Acad Oral Health..

[14] Noh HJ, Kim HE, Kwon HK, Kim BI (2007). Estimate of daily-fluoride intake of powdered milks and baby foods for 6 month old and younger infants in Korea. J Korean Acad Oral Health..

[15] Kim HK, Bae SM, Kho YL, Jung SH (2007). Fluoride ingestion from fluoride toothpaste in preschool children. J Korean Acad Oral Health..

[16] Cardoso VE, Whitford GM, Buzalaf MA (2006). Relationship between daily fluoride intake from diet and the use of dentifrice and human plasma fluoride concentrations. Arch Oral Biol..

[17] Miziara AP, Philippi ST, Levy FM, Buzalaf MA (2009). Fluoride ingestion from food items and dentifrice in 2–6-year-old Brazilian children living in a fluoridated area using a semiquantitative food frequency questionnaire. Community Dent Oral Epidemiol..

[18] Murakami T, Narita N, Nakagaki H, Shibata T, Robinson C (2002). Fluoride intake in Japanese children aged 3–5 years by the duplicate-diet technique. Caries Res..

[19] Jin BH, Ma DS, Moon HS, Paik DI, Hahn SH, Horowitz AM (2003). Early childhood caries: prevalence and risk factors in Seoul, Korea. J Public Health Dent..

[20] Han DH, Kim JB, Park DY (2010). The decline in dental caries among children of different ages in Korea, 2000–2006. Int Dent J..

[21] Lee HJ, Kim JB, Jin BH, Paik DI, Bae KH (2015). Risk factors for dental caries in childhood: a five‐year survival analysis. Community Dent Oral Epidemiol..

[22] Korea Centres for Disease Control and Prevention. Korea National Health and Nutrition Examination Survey. Available at http://knhanes.cdc.go.kr. [Accessed on 15 November 2011].

[23] Ministry of Health and Welfare (2011). Guideline for raw data use of The Fourth Korean National Health and Nutrition Examination Survey (KNHANES IV).

[24] Willet W (1995). Nutritional Epidemiology.

[25] World Health Organization (1997). Oral health surveys: basic methods.

[26] Whitford G (1996). The metabolism and toxicity of fluoride. Monogr Oral Sci.

[27] Taves DR (1968). Separation of fluoride by rapid diffusion using hexamethyldisiloxane. Talanta.

[28] Richards A, Kragstrup J, Josephsen J, Fejerskov O (1986). Dental fluorosis developed in post-secretory enamel. J Dent Res..

[29] Fejerskov O, Thylstrup A, Larsen MJ (1977). Clinical and structural features and possible pathogenic mechanisms of dental fluorosis. Scand J Dent Res..

[30] Chowdhury NG, Brown RH, Shepherd MG (1990). Fluoride intake of infants in New Zealand. J Dent Res..

[31] Zohouri FV, Rugg-Gunn AJ (2000). Total fluoride intake and urinary excretion in 4-year-old Iranian children residing in low-fluoride areas. Br J Nutr..

[32] Burt BA, Eklund SA (2005). Fluoride: human health and caries prevention. Dentistry, dental practice and the community.

[33] The Korean Nutrition Society (2009). Food Value. Second revision.

[34] Levy SM, Kirisy MC, Warren JJ (1995). Sources of fluoride intake in children. J Public Health Dent..

[35] The Korean Nutrition Society (2010). Dietary reference intakes for Koreans.

[36] Warren JJ, Levy SM, Broffitt B, Cavanaugh JE, Kanellis MJ, Weber-Gasparoni K (2009). Considerations on optimal fluoride intake using dental fluorosis and dental caries outcomes-a longitudinal study. J Public Health Dent..

[37] Abuhaloob L, Maguire A, Moynihan P (2015). Total daily fluoride intake and the relative contributions of foods, drinks and toothpaste by 3- to 4-year-old children in the Gaza Strip – Palestine. Int J Paediatr Dent..

[38] Kim JY, Lee JH, Kim EK, Kim JB (2003). User rate of fluoride-containing toothpaste in Ulsan metropolitan city. J Korean Acad Oral Health..

